# Gender Difference in Gender Bias: Transcranial Direct Current Stimulation Reduces Male’s Gender Stereotypes

**DOI:** 10.3389/fnhum.2019.00403

**Published:** 2019-11-22

**Authors:** Siqi Wang, Jinjin Wang, Wenmin Guo, Hang Ye, Xinbo Lu, Jun Luo, Haoli Zheng

**Affiliations:** ^1^School of Economics, Zhejiang University, Hangzhou, China; ^2^School of Economics, Zhejiang University of Finance and Economics, Hangzhou, China; ^3^Center for Economic Behavior and Decision-making (CEBD), Neuro & Behavior EconLab (NBEL), Zhejiang University of Finance and Economics, Hangzhou, China; ^4^Interdisciplinary Center for Social Sciences (ICSS), Zhejiang University, Hangzhou, China

**Keywords:** gender stereotypes, medial prefrontal cortex, transcranial direct current stimulation, implicit associations test, gender difference

## Abstract

Stereotypes exist in the interactions between different social groups, and gender stereotypes are particularly prevalent. Previous studies have suggested that the medial prefrontal cortex (mPFC) is involved in the social cognition that plays an important role in gender stereotypes, but the specific causal effect of the mPFC remains controversial. In this study, we aimed to use transcranial direct current stimulation (tDCS) to identify a direct link between the mPFC and gender bias. Implicit stereotypes were measured by the gender implicit association test (IAT), and explicit prejudice was measured by the Ambivalent Sexism Inventory (ASI). We found that male and female participants had different behavioral and neural correlates of gender stereotypes. Anodal tDCS significantly reduced male participants’ gender D-IAT scores compared with cathodal and sham stimulation, while the stimulation had an insignificant effect in female participants. The reduction in male participants’ gender bias mainly resulted from a decrease in the difference in reaction time (RT) between congruent and incongruent blocks. Regarding the explicit bias measurement, male and female participants had distinct attitudes, but tDCS had no effect on ASI. Our results revealed that the mPFC played a causal role in controlling implicit gender stereotypes, which is consistent with previous observations and complements past lesion, neuroimaging, and transcranial magnetic stimulation (TMS) studies and suggests that males and females have different neural bases for gender stereotypes.

## Introduction

Stereotypes refer to socially shared conceptual attributes associated with members of a social category that describe their traits and characteristics (Greenwald and Banaji, 1995; Amodio, 2014). On the one hand, this automatic association process strengthens the distinction of different groups through overgeneralized social categorization, which is efficient as a cognitive heuristic for simplifying the complexity of the physical and social world (Abrams and Hogg, 1988); on the other hand, it influences people’s social attitudes and behavior, which leads to prejudices, discrimination, and more severe social conflicts (Amodio, 2014). Gender stereotypes have appeared in the mass media and the general public, have been described and discussed in the research literature (Gray, 1992; Rudman et al., 2001), have attracted the attention of both males and females, and have contributed to the foundation of beliefs and behaviors in terms of gender (Becker and Sibley, 2009). In part, gender stereotypes reflect the different characteristics of genders; however, the broad generalization of such a large group of people can never be true and accurate. For example, although social gender stereotypes accentuate gender differences, males and females are more similar than different on most but not all psychological variables (Hyde, 2005). In addition, the intensity of gender stereotypes and the perception of similarities and differences in characteristics between males and females vary across cultures (Guimond, 2008).

Explicit measures are commonly used for assessing an individual’s stereotypes and bias towards a particular group, and these measures require participants to report their own attitudes (Olson and Zabel, 2009). Studies using explicit measures have shown that levels of stereotyping and sexism have reduced in the past few years, but these specious conclusions were drawn from women more than from men (Spence and Buckner, 2000) and cannot reflect unconscious bias when controlled and regulated by social norms and political correctness (Rudman et al., 2001). Moreover, old-fashioned sexist beliefs have gradually evolved from the appearance of discriminatory behavior and negative beliefs towards women to modern sexism (Swim et al., 1995) and neosexism (Tougas et al., 1995) and have been expressed under subtle guises, such as ambivalence and chivalry (Glick and Fiske, 1996; Barreto and Ellemers, 2005).

Implicit measures on gender stereotypes have developed during the past few decades (Rudman and Kilianski, 2000; Rudman et al., 2001); however, previous studies have also revealed that the correlations between results from explicit and implicit methods vary across studies (Greenwald and Banaji, 1995; Rudman and Kilianski, 2000), which has led to a further discussion of the power of implicit measures. The implicit association test (IAT) is one of the most popular methods consistently used for measuring the automatic concept–attribute associations that underlie implicit social biases and stereotypes (Greenwald et al., 1998). In gender stereotypes, this method assesses the association of a target name (either a male name or a female name) with respective attribute categories (e.g., strong vs. weak) that represent the social stereotypes towards these different groups of people. This task requires participants to categorize the target names and attribute words by pressing two corresponding response keys as quickly as possible when they see the words appear on the computer screen. In congruent blocks, participants are instructed to categorize male names and strong attributes using one response key, while female names and weak attributes are categorized by pressing another key. In incongruent blocks, the response mapping is reversed, so male names and weak attributes share one key, and female names and strong attributes share the other key. Since the response time and accuracy rate in the congruent blocks are different from what is obtained in the incongruent blocks, the IAT scores can be calculated following a standard procedure (Greenwald et al., 2003), which represents an individual’s personal implicit social bias towards these two genders.

Gender stereotypes were discovered in past behavioral research, and males and females tend to have different patterns of evaluative gender stereotypes (Rudman et al., 2001). Recently, Pavlova et al. (2014) conducted a series of experiments manipulating implicit and explicit gender stereotyping information and identified the susceptibility to these attitudes. Messages delivered in explicit positive (implicit negative) terms and explicit negative (implicit positive) terms can elicit significant gender differences in cognitive performance on a task with no initial gender gap, and this gender effect is more pronounced in females. However, these studies still lacked direct neural evidence underlying the fluctuation in gender bias.

In accordance with behavioral research, recent neurocognitive studies have investigated the neural basis of prejudice and stereotypes and have found that these psychological phenomena primarily rely on the function of a specific brain region, the medial prefrontal cortex (mPFC; Amodio, 2014). In the social cognition context, the mPFC is associated with the ability to “mentalize,” which underlies theory of mind, and furthermore, with the formation of impressions about other people (Frith and Frith, 1999; Amodio and Frith, 2006; Amodio, 2014), and the mPFC is more activated during the judgment of people than in the judgment of inanimate objects (Mitchell et al., 2002). Neural activity within the mPFC also predicted empathy and an altruistic motivation towards ingroup members (Mathur et al., 2010; Cikara et al., 2011a), while the absence of activity of the mPFC was observed in the “dehumanization” process towards outgroup members (Harris and Fiske, 2006), which leads to biased attitudes and discrimination. In a gender prejudice study, mPFC activation in men who had stronger hostile sexist attitudes when viewing sexualized images of female bodies was lower than that in men who had weaker attitudes (Cikara et al., 2011b), which demonstrates the neural function of the mPFC in sexual objectification. These studies revealed a correlation of prejudice and mPFC activity in the gender field but did not provide direct evidence that proved a causal relationship.

Compared with the role of the mPFC in prejudice, it is more directly involved in stereotyping (Amodio, 2014). According to previous studies, the mPFC participated in brain functions related to stereotypes, for example, cognitive control (Amodio and Frith, 2006), automatic associations, storing social knowledge (Mitchell et al., 2002; Krueger et al., 2009), and integrating information to coordinate social behavior (Contreras et al., 2012; Gilbert et al., 2012). However, the function of the mPFC in gender stereotypes is still not clear. Neuroimaging studies have demonstrated the critical role of the mPFC in gender stereotyping using the behavioral task IAT. Using functional magnetic resonance imaging (fMRI), the anteromedial PFC was significantly activated in congruent blocks of the gender IAT, where the association between gender and social attributes was consistent with the stereotypes (Knutson et al., 2007). Quadflieg et al. (2009) also found that the ventromedial prefrontal cortex (VMPFC) shows stronger activation for stereotypic judgments than for non-stereotypical judgments, which further confirms the indispensable role of the mPFC. However, the results of lesion studies on the function of the mPFC are not all consistent. In earlier clinical observations, male patients with VMPFC lesions had a lower level of gender stereotypes than patients with dorsolateral prefrontal cortex (DLPFC) lesions (Milne and Grafman, 2001). However, a subsequent clinical study found that large lesions in the VMPFC increased stereotypical attitudes (Gozzi et al., 2009). The divergent conclusions result from the different classifications of the brain-damaged region: in Milne and Grafman (2001), patients had damage to both lateral and medial sectors of the ventral PFC, but in Gozzi et al. (2009), the researchers distinguished participants who had lesions in these regions and suggested a differential function of these two sectors of the mPFC.

Although neuroimaging and lesion studies demonstrated associations between the mPFC and gender stereotypes, the direct causal relationship remained imprecise. Brain stimulation technologies such as transcranial magnetic stimulation (TMS) and transcranial direct current stimulation (tDCS) can modulate the activity of target brain regions and establish causal connections between the brain and decisions. One TMS study, Cattaneo et al. (2011) found that applying TMS over the right anterior dorsomedial prefrontal cortex (aDMPFC) of male participants led to increased gender stereotypes as assessed by the IAT. In the present study, we aimed to investigate the effect of tDCS on the mPFC of subjects performing a gender stereotyping task. We chose to apply this noninvasive brain stimulation technique because of the features and advantages compared with TMS (Nitsche and Paulus, 2001; Fecteau et al., 2007). tDCS is safe and easy to use with reliable modulatory effect (Nitsche and Paulus, 2001). Moreover, tDCS does not cause noise interference, nor does it cause muscle twitching during stimulation, which makes it a good choice for performing the IAT, which needs a rapid response (Sellaro et al., 2016). In addition, tDCS can apply reliable sham stimulation, which produces a similar skin sensation but does not modulate the excitability of the brain region (Gandiga et al., 2006; Sellaro et al., 2016). More importantly, tDCS can both enhance and suppress the excitability of local brain activity (Nitsche and Paulus, 2001; Ardolino et al., 2005), so we can determine whether tDCS applied over the mPFC changed the participant’s gender stereotypes and thereby figured out the precise causal role of the mPFC in this process, which would also provide complementary evidence for the TMS study. Furthermore, to test the possible explicit prejudice of the participants influenced by tDCS, we investigated whether there were any divergent results between conscious and unconscious attitudes.

## Materials and Methods

### Participants

A total of 192 right-handed healthy students (96 males, mean age = 20.53 SD = 2.00; 96 females, mean age = 20.21, SD = 1.58) participated in our experiments. All of the participants declared no history of psychiatric illness or psychiatric problems, had normal or corrected-to-normal vision, and were naïve to tDCS, our decision-making task, and IAT. Before participants started the tasks, all of them gave written informed consent approved by the Zhejiang University ethics committee. The experiment lasted approximately one and a half hours, and each participant received an average payment of 30 RMB yuan (approximately 4.35 United States dollars) after the experiment. No participants reported any adverse side effects regarding pain in the scalp or headaches after the experiment.

### tDCS

tDCS applied a weak direct current to the scalp *via* two saline-soaked surface sponge electrodes (5 cm × 7 cm; 35 cm^2^). The current was constant and was delivered by a battery-driven stimulator (NeuroConn, Ilmenau, Germany). It was adjusted to induce cortical excitability of the target area without any physiological damage to the participants. Various orientations of the current had various effects on cortical excitability. In general, anodal stimulation would enhance cortical excitability, whereas cathodal stimulation would restrain it (Nitsche and Paulus, 2000).

**Figure 1:**
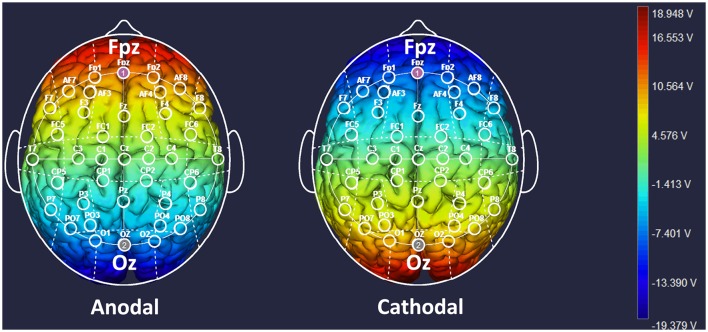
Locations of the electrodes and stimulation modes in transcranial direct current stimulation (tDCS) treatments. Schematic of electrode positions Fpz and Oz based on the international electroencephalography (EEG) 10–20 system of the human brain. The shading represents the range of input voltage from −19.379 V to 18.948 V.

Participants were randomly assigned to one of three tDCS treatments, and the target areas were localized according to the International electroencephalography (EEG) 10–20 System. For anodal stimulation over mPFC (*n* = 64, 32 males and 32 females), the anodal electrode was placed horizontally over the Fpz position, whereas the return electrode was placed horizontally over Oz (Sellaro et al., 2015). For the cathodal stimulation (*n* = 64, 32 males and 32 females), the polarity was reversed, where the cathodal electrode was placed over Fpz, whereas the anodal electrode was placed over Oz (Figure 1). The current was constant for 20 min and was 1.5 mA in intensity, with a 30 s ramp up and down; the safety and efficiency of this stimulation have been demonstrated in previous studies (Riva et al., 2015). For sham stimulation (*n* = 64, 32 males and 32 females), the procedures were the same as in the active tDCS, but the stimulation was automatically turned off after 30 s without the participant’s knowledge. The participants may have felt the initial itching, but there was no current for the rest of the stimulation. This method of sham stimulation has been shown to be reliable (Gandiga et al., 2006). Before the decision-making tasks, the laboratory assistant put a tDCS device on the participant’s head for stimulation. After 20 min of stimulation, the tDCS device was taken off, and the participant was then asked to complete several tasks.

### Task and Procedure

All of the participants received a single-blinded stimulation session (either anodal, cathodal, or sham stimulation), with tDCS applied on the mPFC for 20 min, and then completed IAT tasks programmed by Inquisit 4 (Millisecond Software, Seattle, WA, USA). After the IAT task, they were asked to complete a questionnaire including an explicit test and personal information.

In the IAT task, 20 words were used as stimuli—10 common typical Chinese names and 10 attributes. Five of the names were Chinese male names, and five were Chinese female names. The attribute words consisted of five strong words, and five weak words, which were selected from a previous gender stereotyping study (Rudman et al., 2001); the stimuli from that study have been applied several times in other neurological studies using gender IAT since then (Knutson et al., 2007; Gozzi et al., 2009; Cattaneo et al., 2011).

The task used the procedure designed by Greenwald et al. (1998) consisting of five blocks. Blocks 1, 2, and 4 are for practice, and the others (Blocks 3 and 5) are test blocks. In (practice) Block 1, participants were asked to classify male and female names by pressing left (E) and right keys (I). In (practice) Block 2, they were then asked to categorize the strong words and weak words using these two keys as well. In (practice) Block 4, participants were asked to categorize male and female names again, but the key assignments were reversed compared with Block 1. In (test) Block 3 and Block 5, names and attributes words are combined. One of these blocks was in the congruent condition, where participants were required to press key “E” for male names with the left hand and strong words and key “I” for female names and weak words with the right hand. The other block was in the incongruent condition, and the association was switched such that female names shared key “E” with strong words, and male names shared key “I” with weak words. The order of the congruent and incongruent blocks was counterbalanced; meanwhile, the order of the name practice blocks corresponded with the order of the test blocks (the position of Block 1 was swapped with Block 4 when Block 3 was incongruent and Block 5 was congruent).

Stimuli were presented in the center of the computer screen in white text on a black background using Inquisit 4 (Millisecond Software, Seattle, WA, USA). The category labels (“men” and “women, ” “strong” and “weak”) were displayed on the left and right top sides of the screen. Practice Blocks 1, 2, and 4 had 20 trials, and test Blocks 3 and 5 had 40 trials. To complete the task, Participants needed to classify names and attributes words by pressing the keys “E” and “I” on the computer keyboard according to the label’s position. Each trial was kept on the screen until the participant had given the correct response, followed by a 500 ms blank screen. Participants were asked to respond as quickly and accurately as possible when stimuli appeared on the screen.

### Analysis

The critical variables are mean reaction time (RT) and percentage of error rate (PE), which reflect the subjects’ direct responses to the different types of associations. According to Greenwald et al. (2003), an improved algorithm performed better in measuring implicit association strength, so we calculated D-IAT scores for the three stimulation conditions following this procedure. All trials except the extreme long trials (latencies >10,000 ms) were included, and error latencies were replaced with block mean latencies plus 600 ms. The RTs and PEs were then synthesized to D-IAT scores—the difference between the adjusted latencies of the incongruent and congruent blocks divided by the pooled standard deviation of all trials. In general, these three variables together indicate how strong the stereotypes are, with higher IAT scores and larger differences in RTs and PEs between the congruent block and the incongruent block representing a stronger implicit bias towards males or females.

To evaluate explicit stereotypes, we used the Ambivalent Sexism Inventory (ASI; Glick and Fiske, 1996) and calculated the scores according to the standard method (Supplementary Table S1). Because some of the reversed-worded items did not perform well when translated into other languages in cross-cultural studies (Glick et al., 2000), only valid items were retained in the test. In general, the ASI scores represent ambivalent attitudes towards women. HS represents hostile sexism, expressing negative stereotypes and attitudes towards women, while BS represents benevolent sexism expressing positive stereotypes and attitudes, both of which complementarily generate gender inequity in various cross-cultural ideologies (Glick and Fiske, 2001). In addition, there are three subfactors of benevolent sexism: protective paternalism, complementary gender differentiation, and heterosexual intimacy, which correspond with three types of questions (BP, BG, and BI) in BS.

## Results

The data were statistically evaluated using SPSS software (version 22, SPSS Inc., Chicago, IL, USA). The significance level was set at 0.05 for all analyses.

### Implicit Measures

**Figure 2:**
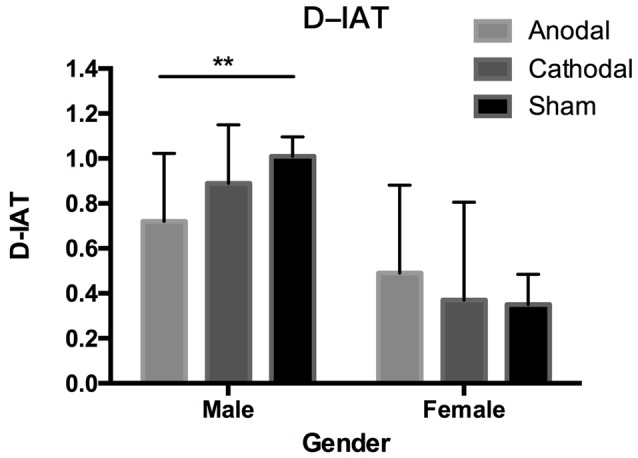
Data of D-implicit association test (IAT) scores. Error bars indicate 95% confidence intervals. Asterisks indicate significant differences in gender stereotypes between treatments.

The Shapiro–Wilk test showed that the residuals of the D-IAT scores were normally distributed (*p* = 0.129). To test whether both male and female participants had gender stereotypes, we used a one-sample *t*-test to compare D-IAT scores and zero. Figure 2 shows both male and female participants’ IAT-scores in different stimulation conditions. In all of the male data, there was a significant difference between the IAT-D scores and zero (*t*_(95)_ = 29.35, *p* < 0.001, Mean = 0.87, SD = 0.29). Analyses also showed that IAT-D scores from all three stimulation conditions were significantly different from zero respectively (anodal: *t*_(31)_ = 13.47, *p* < 0.001, Mean = 0.72, SD = 0.30; cathodal: *t*_(31)_ = 19.30, *p* < 0.001, Mean = 0.89, SD = 0.26; sham: *t*_(31)_ = 23.73, *p* < 0.001, Mean = 1.01, SD = 0.24), which indicated that male subjects had strong associations of male names with strong attributes and female names with weak attributes regardless of the stimulation conditions. As for the female subjects in the three tDCS types, they also had gender stereotypes in all groups. *t*-tests revealed that the D-IAT scores from the three stimulation conditions were significantly different from zero respectively (all: *t*_(95)_ = 9.86, *p* < 0.001, Mean = 0.40, SD = 0.40; anodal: *t*_(31)_ = 7.03, *p* < 0.001, Mean = 0.49, SD = 0.40; cathodal: *t*_(31)_ = 4.81, *p* < 0.001, Mean = 0.37, SD = 0.44; sham: *t*_(31)_ = 5.36, *p* < 0.001, Mean = 0.35, SD = 0.37).

One-way ANOVA performed on the D-IAT scores of all subjects using gender and tDCS types as factors showed that the main effect of gender (*F*_(1,186)_ = 90.34, *p* < 0.001, $\eta_{\text{p}}^{2}$ = 0.32) and the interaction of gender and stimulation conditions (*F*_(2,186)_ = 6.22, *p* = 0.002, $\eta_{\text{p}}^{2}$ = 0.06) were significant, which indicated that male and female participants’ D-IAT scores have different patterns. The main effect of tDCS types was not significant (*F*_(2,186)_ = 0.85, *p* = 0.429, $\eta_{\text{p}}^{2}$ < 0.01). *Post hoc* analysis using Bonferroni corrections revealed that male participants’ D-IAT scores significantly decreased when subjects underwent anodal stimulation compared with sham stimulation (*p* = 0.003), while the D-IAT scores of those that underwent cathodal stimulation were not significantly changed compared with the sham group (*p* = 0.476) and anodal group (*p* = 0.163). However, the female participants’ D-IAT scores were not significantly changed by the stimulation. Bonferroni *post hoc* tests revealed that no significant result was found in the pairwise comparison between these three stimulations (*p* > 0.1). *Post hoc* analysis using Bonferroni corrections also found that male participants’ D-IAT scores were higher than those of female participants in all stimulation conditions (anodal: *p* = 0.006, cathodal: *p* < 0.001, and sham: *p* < 0.001).

**Figure 3:**
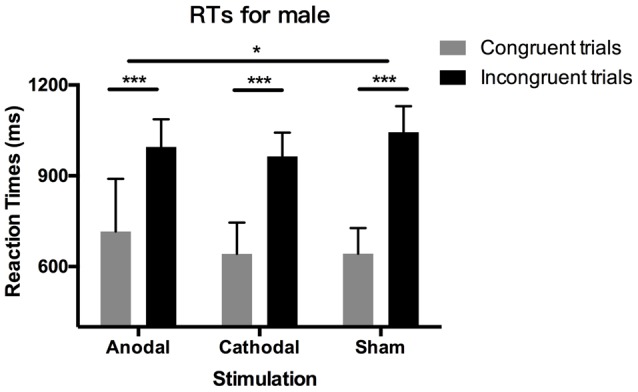
Data on reaction times (RTs) in male participants. Error bars indicate 95% confidence intervals. Asterisks within stimulation conditions indicate significant differences in RTs between congruent and incongruent blocks. Asterisks between stimulation conditions indicate significant differences in the gap of congruent and incongruent blocks between stimulations.

D-IAT scores depend on RTs and PEs: lower D-IAT scores mean higher RTs and PEs in the congruent blocks or lower RTs and PEs in the incongruent blocks. Therefore, we further decomposed the D-IAT effect and analyzed RTs and PEs. Table 1 shows all the means and SD for RTs and PEs across genders, blocks, and stimulation conditions. The Shapiro–Wilk test showed that the residuals of RTs and PEs in congruent and incongruent blocks were not normally distributed (*p* < 0.05), so we performed non-parametric tests to analyze them. First, we tested RTs; Figure 3 shows the results. The Wilcoxon signed-rank test showed significant results for the relationship between RTs in congruent blocks and incongruent blocks (*p* < 0.001), indicating that RTs were overall higher in incongruent blocks than in congruent blocks. This difference is RTs remained present for both male and female participants in the three stimulation conditions (*p* < 0.001).

**Table 1:** Mean and SD D-IAT scores, reaction times, percent of error, and rate correct scores across genders, blocks, and stimulations.

Gender	Stimulation	Anodal		Cathodal		Sham		Average	
	Block	Cong	Incong	Cong	Incong	Cong	Incong	Cong	Incong
Male	RT (ms)	715.98***	994.82***	641.77***	963.66***	642.43***	1043.85***	666.73^†^	1000.78^†††^
	(SD)	173.80	254.12	103.03	218.71	84.85	238.73	129.99	237.45
	PE (%)	1.80**	4.22**	2.42***	5.94***	1.88***	7.42***	2.03^†^	5.86^††^
	(SD)	2.13	4.51	3.39	5.30	2.77	8.51	2.79	6.42
	D_IAT	0.72		0.89		1.01		0.87
	(SD)	0.30		0.26		0.24		0.29
	RCS	1.43***	1.01***	1.56***	1.03***	1.55***	0.94***	1.51^††^	0.99^†††^
	(SD)	0.27	0.21	0.24	0.24	0.19	0.24	0.24	0.23
Female	RT (ms)	694.95***	875.25***	682.36***	816.22***	713.32***	856.9***	696.88^†^	849.45^†††^
	(SD)	107.18	195.55	124.36	251.52	116.46	191.51	115.69	213.79
	PE (%)	2.89	3.59	3.28	4.30	2.34	3.28	2.84^†^	3.72^††^
	(SD)	3.31	3.42	3.89	4.85	2.69	4.64	3.32	4.32
	D_IAT	0.49		0.37		0.35		0.40
	(SD)	0.39		0.44		0.37		0.40
	RCS	1.43***	1.16***	1.46***	1.26***	1.40***	1.18***	1.43^††^	1.20^†††^
	(SD)	0.21	0.26	0.25	0.30	0.22	0.24	0.23	0.27

*Asterisks indicate statistically significant differences between congruent and incongruent blocks. Daggers indicate statistically significant differences between male and female genders*.

More importantly, we analyzed the factors of block conditions, gender, and stimulation conditions. First, we applied these tests on the data from male participants separately. With a Kruskal–Wallis test, we found no significant difference in RTs either in congruent blocks or in incongruent blocks (*p* > 0.1), indicating that tDCS did not change the latencies in these two distinctive blocks respectively. However, the difference in RT between congruent blocks and incongruent blocks was significantly modulated by tDCS (*p* = 0.028). *Post hoc* analysis using Dunn–Bonferroni corrections revealed that the difference in RTs in anodal stimulation is significantly smaller than that in sham stimulation (*p* = 0.023), while the cathodal stimulation had no significant effect compared with the anodal and sham group (*p* > 0.1). These results indicated that the effect of tDCS on male participants’ gender stereotypes stemmed from the relative association between congruent blocks and incongruent blocks rather than these two blocks independently. As for female participants, the effect of tDCS disappeared. Kruskal–Wallis tests on RTs in congruent blocks, incongruent blocks, and the difference of them were all insignificant (*p* > 0.1).

**Figure 4:**
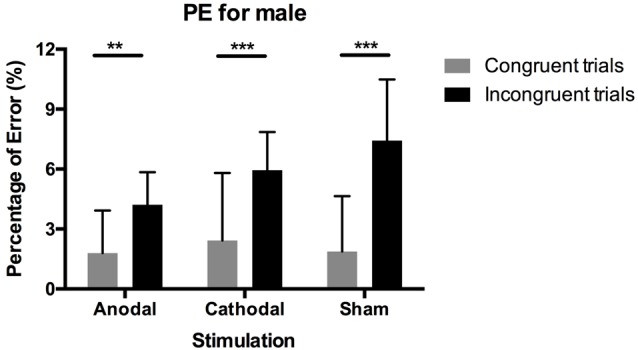
Data on percentage of error in male participants. Error bars indicate 95% confidence intervals. Asterisks within stimulation conditions indicate significant differences in PEs between congruent and incongruent blocks.

We also tested PEs using the same non-parametric test. Figure 4 shows the results. The Wilcoxon signed-rank test showed a significant difference between PEs in congruent blocks and incongruent blocks (*p* < 0.001), indicating that participants made more mistakes overall in incongruent blocks than in congruent blocks. For male participants in the three stimulation conditions, this difference in PEs between block conditions still existed (cathodal, sham: *p* < 0.001, anodal: *p* = 0.002), while differences in female participants’ error rate were insignificant between condition blocks. Meanwhile, for both males and females, the Kruskal–Wallis test on PEs in both congruent blocks and incongruent blocks and the difference between them were all insignificant (*p* > 0.1).

We further focused on gender differences in the RTs and PEs of both congruent and incongruent blocks. The Mann–Whitney test was applied to show that the differences in RTs and PEs between genders existed in both block conditions, but the differences were in the opposite direction between block conditions. As for RTs, in incongruent blocks, male participants reacted significantly more slowly than females (*p* < 0.001) but in congruent blocks, male participants reacted significantly more quickly (*p* = 0.014). In terms of PEs, males made significantly more mistakes than females in incongruent blocks (*p* = 0.010), but in congruent blocks, males made significantly fewer mistakes (*p* = 0.049).

### Explicit Measures

The Shapiro–Wilk test showed that the residuals of ASI were normally distributed (*p* = 0.116). To investigate whether tDCS directly changes explicit prejudice, we tested the effect of tDCS on the explicit test (Table 2). One-way ANOVA with stimulation conditions (anodal, cathodal, and sham) and gender (male and female) as between-subjects factors showed no significant main effect in stimulation conditions (*F*_(2,186)_ = 2.46, *p* = 0.088, $\eta_{\text{p}}^{2}$ = 0.03), gender (*F*_(1,186)_ = 3.46, *p* = 0.064, $\eta_{\text{p}}^{2}$ = 0.02), and the interaction of stimulation conditions and gender (*F*_(2,186)_ = 0.20, *p* = 0.818, $\eta_{\text{p}}^{2}$ < 0.01), which revealed that tDCS did not modulate the explicit gender stereotypes of either male or female participants.

**Table 2:** Mean and SD of Ambivalent Sexism Inventory scores.

Gender		HS	BS	BP	BG	BI	ASI
Male	Mean	2.35	2.55	3.03	1.69	3.12	2.45
	(SD)	0.77	0.80	0.98	0.92	1.42	0.64
Female	Mean	2.13	2.4	2.65	2.29	2.17	2.26
	(SD)	0.84	0.92	1.08	0.93	1.55	0.74

Since the ASI has four subscales: HS, BI, BG, and BP, and only the residuals of HS were normally distributed according to the Shapiro–Wilk test (HS: *p* = 0.152 BI, BG, BP: *p* < 0.05), we performed non-parametric tests for analysis. Kruskal–Wallis was used to test the tDCS effect on these subscales from male and female participants, respectively. Overall, tDCS stimulation did not change the subscales (*p* > 0.1). The only explicit attitude influenced by tDCS was BG from female participants (*p* = 0.045). *Post hoc* analysis using Dunn–Bonferroni corrections revealed that anodal stimulation reduced the intensity of females’ attitudes on gender differentiation compared with sham stimulation (*p* = 0.041).

The Mann–Whitney test was applied to analyze the gender difference in the subscales further. There were some distinctions between the attitudes of male and female participants. On the whole, males and females had a similar degree of hostile sexism towards the female (*p* = 0.083). However, there were significant differences in benevolent sexism between male and female participants. Males had higher BI factor values than females (*p* < 0.001), and they also had higher BP factor values (*p* = 0.027). Nevertheless, the male participants’ sexism was weaker than that of females in terms of the BG factor (*p* < 0.001). These results demonstrated that males and females had their own reasons for benevolent sexism: males are more sexist in terms of protective paternalism and heterosexual intimacy, while females focus more on complementary gender differentiation.

### Correlation Between Implicit and Explicit Measures

Finally, we tested whether the explicit attitudes, the ASI scores, were correlated with the implicit gender stereotypes. The ASI scores were positively correlated with the D-IAT score in the sham group (*ρ* = 0.24, *p* = 0.053 in a Pearson correlation test). In our study, the HS from our participants in the sham situation had a significant relationship with the D-IAT score according to Pearson correlation test (*ρ* = 0.39, *p* = 0.002 for HS, *ρ* = 0.03, *p* = 0.83 for BS), which implied that hostile sexism was the only explicit attitude correlate with the implicit gender stereotypes. When we tested the correlation of all six (three stimulation conditions × two genders) combinations, HS was only positively correlated with the D-IAT scores for both males (*ρ* = 0.34, *p* = 0.057) and females in the sham stimulation (*ρ* = 0.34, *p* = 0.061), a trend close to significance.

### Robustness Analysis: RCS

**Figure 5:**
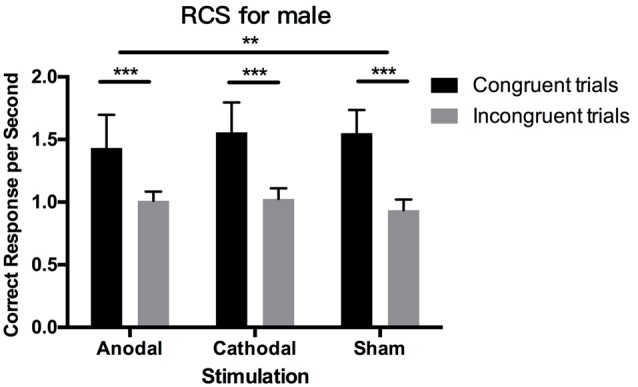
Data on RCS in male participants. Error bars indicate 95% confidence intervals. Asterisks within stimulation conditions indicate significant differences in RCS between congruent and incongruent blocks. Asterisks between stimulations indicate significant differences in the gap of congruent and incongruent blocks between stimulations.

In the implicit measures, we first calculated D-IAT scores, and then analyzed the RTs and PEs separately. In this section, we further applied another method called the rate correct score or RCS, which combines speed and accuracy as a robustness analysis. The RCS is the number of correct responses divided by the sum of all RTs in the congruent and incongruent conditions, respectively (Woltz and Was, 2006; Vandierendonck, 2017). Table 1 shows all of the mean and SD values for RCS across genders, blocks, and stimulation conditions. Figure 5 also shows the results.

The Shapiro–Wilk test showed that the residuals of RCS in incongruent blocks were normally distributed (*p* = 0.315), while those in congruent blocks were not normally distributed (*p* = 0.027), so we performed non-parametric tests to analyze them. The Wilcoxon signed-rank test showed significant results between RCS in congruent blocks and incongruent blocks (*p* < 0.001), indicating that RCS were higher overall in congruent blocks than in incongruent blocks and that people made more correct responses per second. For both male and female participants in the three stimulation conditions, these differences in RCS between block conditions still existed (*p* < 0.001).

Kruskal–Wallis testing on RCS in both the congruent blocks and incongruent blocks from male and female participants showed that tDCS did not change the RCS in these two distinctive blocks, respectively (*p* > 0.1). However, for male participants, the difference in RCS between congruent blocks and incongruent blocks was significantly modulated by tDCS (*p* = 0.003). *Post hoc* analysis using Dunn–Bonferroni corrections revealed that the difference in RCS in anodal stimulation is significantly smaller than that in sham stimulation (*p* = 0.002), while cathodal stimulation had no significant effect compared with the anodal and sham groups (*p* > 0.1). These results demonstrated that tDCS modulated male participants’ relative correct responses per second between congruent blocks and incongruent blocks, which was consistent with the results of the RT analysis. For female participants, these effects were all insignificant.

We also checked the gender difference in RCS in congruent and incongruent blocks by using the Mann–Whitney test. In congruent blocks, male participants’ number of correct responses per second were higher than females’ (*p* = 0.006), but in incongruent blocks, male participants made significantly fewer correct responses per second than females (*p* < 0.001). This result is consistent with our finding for RTs and PEs, in that differences in RCS between genders existed in both block conditions but the differences were in the opposite direction in the two block conditions.

## Discussion

This article investigated the contribution of the mPFC to stereotypes, specifically within the domain of gender stereotypes, and this effect was found to be limited to male participants. Previous lesion studies (Milne and Grafman, 2001; Gozzi et al., 2009), neuroimaging studies (Knutson et al., 2007; Quadflieg et al., 2009), and a TMS study (Cattaneo et al., 2011) suggested that the mPFC was involved in prejudice and stereotyping (Amodio, 2014), especially in the gender stereotyping assessed by the IAT (Greenwald et al., 1998, 2003). Nevertheless, the mechanistic role of the mPFC in this test remained vague, and the conclusions have not been convergent.

Because of the inconsistency of previous results and the lack of a test of the causal relationship between the mPFC and gender stereotypes, in this study, we applied tDCS over the mPFC in our participants to directly modulate this brain region and reveal the precise effect on gender stereotypes. We found that, when enhancing the activity of the mPFC, the implicit gender stereotyping attitudes of male participants, as indicated by the D-IAT scores measured by the gender IAT (Rudman et al., 2001) were reduced compared to the sham group. This observation demonstrated the causal relationship between mPFC activation and gender-stereotyped attitudes. The reduction in the D-IAT scores mainly stemmed from a decrease in the difference in RTs between the incongruent and congruent blocks when participants underwent anodal stimulation over mPFC, which seems to conflict with previous research that the effect of modulating the activity of the mPFC resulted from altered performance only in the incongruent blocks (Sellaro et al., 2015). In this study, the researchers found that enhancing the activation of the mPFC reduced the negative bias towards social outgroups. Thus, the interpretation was that the mPFC was an essential region in self-regulatory and cognitive control in the context of ethnic stereotyping. Cattaneo et al. (2011) suggested that the inhibition of the aDMPFC by TMS led to an increase in gender bias based on an increased error rate in the incongruent blocks. Our findings were not inconsistent with that result because the aDMPFC is involved in the network mediating cognitive control in the DLPFC. In the present study, the target brain region was the mPFC, or the VMPFC specifically, which proved to have a different function than the DMPFC based on a lesion study (Gozzi et al., 2009). This result can also be compared to the outcome from Gladwin et al. (2012), where anodal stimulation of the L-DLPFC only improved the RT in congruent blocks using the IAT about insects and flowers. They found that the function of the L-DLPFC was to influence working memory, which meant that the activation of the L-DPLFC increased the associations in congruent blocks and led to faster RTs but that, in incongruent blocks, this activation of the brain region affected congruent and incongruent associations at the same time.

In this study, several factors contributed to the changes observed in the congruent blocks comparing to incongruent blocks. First, the effect of mPFC activation on congruent blocks also correlates with the role of the mPFC in memory and decision making. According to Euston et al. (2012), when confronted with different contexts, locations, and events, the mPFC takes part in the process of learning and using the associations between these targets to provide the corresponding response. This function in both long-term and short-term memory provides the possible explanation that the activation of the mPFC reduced the association intensity in congruent blocks but had an effect on both congruent and incongruent associations in incongruent blocks, which finally led to a reduction in the bias. Another reason was that gender stereotypes are culture-sensitive. In Western culture and Chinese culture, the history and current situation of social gender stereotypes are not entirely the same. In the meantime, there is a gap in the intensity of the gender stereotypes between these two societies. For example, the D-IAT scores from our experiment were higher than those from the previous study (Cattaneo et al., 2011). The similar, but not identical, cultural background influences an individual’s neural activity, which underlies cognitive functions such as emotional processing, mental attribution, self-representation, and self-awareness (Han and Northoff, 2008), which possibly causes the distinct change in the congruent blocks during anodal stimulation. The reasons above combined can account for the effect of tDCS on the differences in RTs and RCS between incongruent and congruent blocks. Actually, a more precise role of the mPFC in the neural circuit of prejudice and stereotypes can be found by further combining fMRI and tDCS techniques.

We also revealed that cathodal tDCS had no significant effect on the behavior of either the male or female participants compared with the sham group, which was consistent with Sellaro et al. (2015). This result may be because the mPFC is insensitive to cathodal stimulation, which was also investigated in studies of cathodal stimulation of the somatosensory cortex, while anodal stimulation influenced the activity of this brain area (Matsunaga et al., 2004). Another possible explanation also mentioned in previous studies is that the low background level of activity in the mPFC and the high prejudice baseline have a ceiling effect, which limits the influence of cathodal stimulation (Matsunaga et al., 2004; Sellaro et al., 2015).

This study also investigated gender differences in the view of gender bias. Regarding implicit stereotypes, the female participants’ bias was significantly lower than that of the male participants, and only the male participants’ gender stereotypes were significantly affected by the tDCS, which has also been observed in several studies (Rudman et al., 2001; Knutson et al., 2007; Cattaneo et al., 2011). Additionally, the female participants showed a significant gender bias in the IAT, which resulted from a different gender culture baseline between the societies. One possible reason is that the different behaviors of the male and female participants stemmed from different neural stereotype substrates or different sensitivities to activation of the mPFC during tDCS. The female participants had a relatively higher activation level of the mPFC and lower gender bias so that stimulation power was limited, and stimulation could not further reduce the bias. Because explicit attitudes correlated with D-IAT scores for some of the subscales, different neural activities in the male and female participants may both consciously and unconsciously influence the bias.

In terms of the neural substrates of gender difference, Stam et al. (2019) demonstrated that brain structure-personality associations are dependent on sex. Specifically, in some brain regions, there were inverse associations between temperament and regional gray matter volume (GMv) in males and females, and the brain regions related to gender and temperament were non-overlapping. So, the difference in personality between genders has a sex-specific neural basis. In our study, what we found is consistent with Stam et al. (2019): the difference in implicit gender stereotypic attitudes between male and female have a sex-specific association with the target region, the mPFC. We demonstrated the causal relationship between the mPFC and gender stereotypes by modulating the activity of the mPFC. Although personal characteristics, temperaments and stereotypic attitudes are distinct from each other, for example, temperaments are heritable, homogeneous, and stable while stereotypic attitudes can be influenced by culture and evolved in the lifetime (Comings et al., 2000; Stam et al., 2019), sex-specific associations between brain regions and personal traits and attitudes still exist. In summary, our results provided extensive evidence from personality to stereotypes for the neural basis of gender difference.

In this study, the participants’ explicit prejudicial beliefs were measured by the ASI, which has been widely used in previous research (Glick and Fiske, 1996; Milne and Grafman, 2001; Rudman et al., 2001; Knutson et al., 2007; Cattaneo et al., 2011). The ASI scores correlated with the D-IAT scores in the sham group, which indicated that the explicit attitudes of gender stereotypes and the automatic association process were closely related. However, our study demonstrated that tDCS had no effect on the majority of the explicit test. Therefore, we interpret these findings to show that the explicit gender bias in the ASI was consciously controlled according to social norms and discipline, and this bias expression can be controlled by external influences, such as culture and education (Crandall et al., 2002; Cunningham et al., 2004). This cultural background may explain the different results in our research from previous research. For example, Rudman et al. (2001) revealed that both BS and HS significantly correlated with gender potency stereotypes measured by a similar IAT to that used in our experiment, and these findings demonstrated that participants who held both hostile and benevolent sexist attitudes had the same automatic associations between males and potency. However, in our study, only the HS scores in our participants in the sham stimulation condition showed trends close to a significant relationship with the D-IAT scores through the Pearson correlation test, which revealed that hostile sexism was the only explicit behavior related to the implicit gender stereotypes here.

### Limitations

One limitation of this study is that although our findings confirmed that modulating the excitability of the mPFC reduced male participants’ gender stereotypes, the neural circuitry underlying this process cannot be demonstrated by a single experiment. Future studies may focus on other brain regions and discuss the functions of the mPFC within the neural circuit. Moreover, by using this bipolar tDCS montage, whether only the mPFC influences the gender stereotypes or whether both target and return electrodes and the interaction between them influence participant’s behavior together is still unclear. These issues should be considered seriously in further studies. In addition, this study applied a between-subject design to avoid the learning effect, which can also be improved upon in the future.

## Conclusion

Our study revealed that male and female participants had different behavioral performance and neural substrates regarding gender stereotypes. Males had a relatively higher level of gender stereotyping than females, and the mPFC plays a causal role in controlling male participants’ implicit gender stereotypes. Male participants’ implicit bias was significantly restrained by tDCS, but female participants were not significantly influenced. The stimulation did not directly influence the ability to make automatic associations in congruent blocks or to overcome automatically activated gender-biased associations in incongruent blocks but affected the difference between the two blocks. We also found differences in explicit prejudice between male and female, which have both neural and cultural underpinnings.

## Ethics Statement

This study was carried out in accordance with the recommendations of Zhejiang University ethics committee with written informed consent from all subjects. All subjects gave written informed consent in accordance with the Declaration of Helsinki. The protocol was approved by the Zhejiang University ethics committee.

## Author Contributions

SW and HZ designed the experiment, analyzed the data and wrote the manuscript. SW, JW, WG, HY, XL, JL, and HZ performed the experiment, revised the manuscript and finally approved the version to be published. SW drew figures.

## Conflict of Interest

The authors declare that the research was conducted in the absence of any commercial or financial relationships that could be construed as a potential conflict of interest.
