# Supplementary material for: Gender Difference in Gender Bias: Transcranial Direct Current Stimulation Reduces Male’s Gender Stereotypes
Source: Front Hum Neurosci. 2019 Nov 22;13:403. doi: 10.3389/fnhum.2019.00403 (PMC6889476; doi:10.3389/fnhum.2019.00403)
Supplement: Supplementary file 1 [file Table_1.DOCX]

**Ambivalent Sexism Inventory**

Below is a series of statements concerning men and women and their relationships in contemporary society. Please indicate the degree to which you agree or disagree with each statement using the scale below:

| Disagree Strongly | Disagree somewhat | Disagree slightly | Agree slightly | Agree somewhat | Agree Strongly |
| --- | --- | --- | --- | --- | --- |
| 0 | 1 | 2 | 3 | 4 | 5 |

____ 1. No matter how accomplished he is, a man is not truly complete as a person unless he has the love of a woman.

____ 2. Many women are actually seeking special favors, such as hiring policies that favor them over men, under the guise of asking for "equality."

____ 3. Most women interpret innocent remarks or acts as being sexist.

____ 4. Women are too easily offended.

____ 5. Many women have a quality of purity that few men possess.

____ 6. Women should be cherished and protected by men.

____ 7. Most women fail to appreciate fully all that men do for them

____ 8. Women seek to gain power by getting control over men.

____ 9. Every man ought to have a woman whom he adores.

____ 10. Women exaggerate problems they have at work.

____ 11. Once a woman gets a man to commit to her, she usually tries to put him on a tight leash.

____ 12. When women lose to men in a fair competition, they typically complain about being discriminated against.

____ 13. A good woman should be set on a pedestal by her man.

____ 14. Women, compared to men, tend to have a superior moral sensibility.

____ 15. Men should be willing to sacrifice their own well-being in order to provide financially for the women in their lives.

____ 16. Women, as compared to men, tend to have a more refined sense of culture and good taste.
